# IL-1 drives breast cancer growth and bone metastasis *in vivo*

**DOI:** 10.18632/oncotarget.12289

**Published:** 2016-09-27

**Authors:** Ingunn Holen, Diane V. Lefley, Sheila E. Francis, Sarah Rennicks, Steven Bradbury, Robert E. Coleman, Penelope Ottewell

**Affiliations:** ^1^ Academic Unit of Clinical Oncology, Department of Oncology and Metabolism, Mellanby Centre for Bone Research, University of Sheffield, Sheffield S10 2RX, UK; ^2^ Department of Infection Immunity and Cardiovascular Disease, University of Sheffield, Sheffield S10 2RX, UK

**Keywords:** breast cancer, bone metastasis, IL-1B, IL-1R, anakinra

## Abstract

**Background:**

We have recently identified interleukin 1B (IL-1B) as a potential biomarker for predicting breast cancer patients at increased risk for developing bone metastasis. In mouse models, IL-1B and its receptor (IL-1R1) are upregulated in breast cancer cells that metastasise to bone compared with cells that do not. We have now investigated the functional role of IL-1 by blocking IL-1R signalling with the clinically licensed antagonist, anakinra.

**Methodology:**

6-week old female BALB/c mice received a subcutaneous or intra-venous injection of MDA-MB-231-IV or MCF7 cells. Anakinra (1mg/kg/day) or placebo was administered 3 days before (preventative) or 7 days later (treatment). Tumour volume, apoptosis (TUNEL, Caspase 3), proliferation (Ki67) and angiogenesis (CD34, VEGF and endothelin) were analysed. Effects on bone were measured by uCT, and TRAP, P1NP, IL-1B, TNF alpha and IL-6 ELISA.

**Results:**

Anakinra significantly reduced growth of MDA-MB-231-IV tumours in bone from 6.50+/3.00mm^2^ (placebo) to 2.56+/−1.07mm^2^ (treatment) and 0.63+/−0.18mm^2^ (preventative). Anakinra also reduced the number of mice that developed bone metastasis from 90% (placebo) to 40% (treatment) and 10% (preventative). Anti-tumour effects were not confined to bone, subcutaneous tumour volumes reduced from 656.68mm^3^ (placebo) to 160.47mm^3^ (treatment) and 31.08mm^3^ (preventative). Anakinra did not increase tumour cell apoptosis but reduced proliferation and angiogenesis in addition to exerting significant effects on the tumour environment reducing bone turnover markers, IL-1B and TNF alpha.

**Conclusions:**

Our novel data demonstrate a functional role of IL-1 signalling in breast tumour progression and metastasis, supporting that anakinra could be repurposed for the treatment of breast cancer bone metastasis.

## INTRODUCTION

About three quarters of patients that develop metastatic breast cancer will have bone involvement. Metastatic bone disease is incurable with a median survival of ~2-3 years following diagnosis, hence new therapeutic approaches are still needed to improve outcome in this poor prognosis group. We have recently identified IL-1B as a potential prognostic biomarker for early breast cancer patients at increased risk for the subsequent development of skeletal metastases [1]. In a study of 150 patients presenting with stage II /III breast cancer included in a clinical trial (AZURE), we found significant correlations between expression of IL-1B in the primary tumour and both disease recurrence at any site (P < 0.0001) and, specifically, metastasis to bone (P < 0.0001) after median follow up of 84 months. Furthermore, mouse model systems of human breast cancer have provided evidence that increased IL-1B expression is linked with the ability of tumour cells to home to the bone environment [1].

IL-1B expression is elevated in a variety of cancers (including breast, prostate, colon, lung, head and neck cancers and melanomas) and patients with IL-1B producing tumours generally have a worse prognosis [2]. Whereas IL-1B expression has been implicated in metastatic behaviour of breast cancer cells *in vitro* [3–6], no functional *in vivo* studies have been published and no specific link to bone metastasis has been explored. The most compelling evidence for a role of IL-1B in bone metastasis was reported using a mouse model of prostate cancer. In this study, overexpression of IL-1B in non-metastatic prostate cancer cells promoted bone metastasis, whereas knockdown of IL-1B impaired bone progression by metastatic cells [7]. Experimental models of Lewis lung carcinoma, pulmonary adenocarcinoma and squamous adenocarcinoma have shown that local production of IL-1B influences tumour growth and metastasis, either through direct proliferative effects or by promoting inflammatory and angiogenic pathways in surrounding host cells [5, 8–9]. Evidence from giant cell tumour and glioma cells, small cell lung cancer and melanoma indicate that IL-1B induces proliferation of new blood vessels *in vivo* and that these activities are mediated indirectly via IL-1B effects on other cell types, including fibroblasts and immune cells in the tumour microenvironment [10–11]. It is likely that IL-1B directly impacts on tumour aggressiveness and metastatic potential due to its ability to influence multiple molecular pathways, and that blocking IL-1B activity therefore has the potential to be an effective anti-cancer therapy.

IL-1B exerts its activity by binding to the type 1 receptor (IL-1R1) and clinical data from patients with prostate cancer show an association between high levels of IL-1R1 in the primary tumour with poor prognosis, while low levels of IL-1B in the primary tumour and associated stroma predict a good prognosis [12]. Anakinra, a recombinant IL-1 receptor antagonist that binds to IL-1 receptors type I and type II, is currently licenced to treat patients with neonatal onset multi system inflammatory disease and rheumatoid arthritis, and has also recently been used in a Phase 2 clinical trial in patients with myocardial infarction (100 mg/day for 14 days) [13–15]. Trials in over 250,000 patients have shown anakinra to have an excellent safety profile, being well tolerated by patients who report little to no side effects and is not associated with increased susceptibility to opportunistic infections compared with other biological agents [16]. In the current study, bone homing MDA-MB-231 IV cells were injected into the lateral tail vein to model bone metastasis [1] and MDA-MB-231-IV and MCF7 cells were injected subcutaneously to model non-metastatic oestrogen receptor (ER) +ve and ER-ve breast cancer. These models were used to investigate the therapeutic efficacy of a clinically relevant dose of anakinra (1mg/kg/day, equivalent to 100mg/day in people [17]) to explore the potential use as a novel treatment for breast cancer bone metastasis.

## RESULTS

### Effects of IL-1R blockade on tumour growth in bone

**Figure 1:**
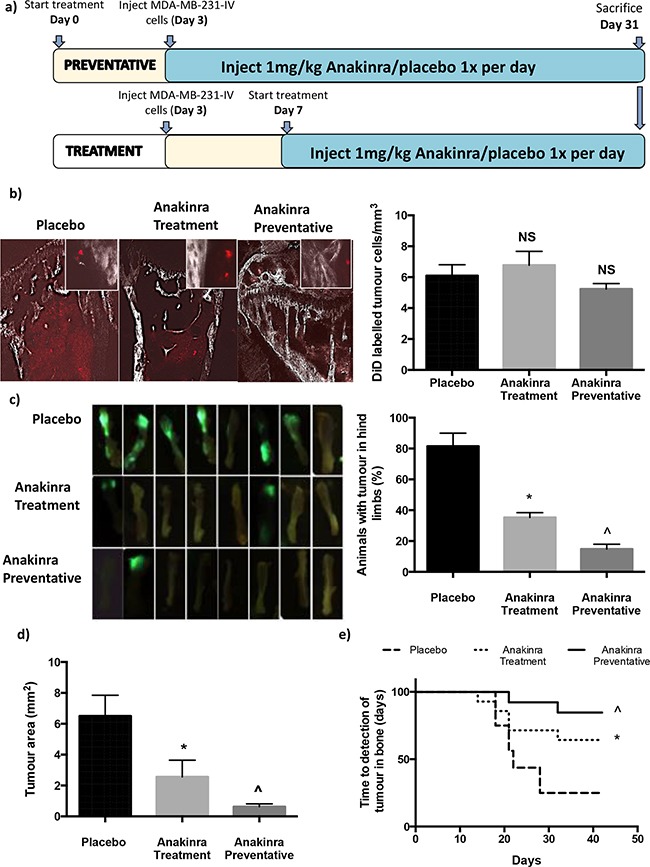
Effects of anakinra on MDA-MB-231 breast cancer dissemination and growth in bone 6-week old BALB/c nude mice were administered 1mg/kg/day anakinra 3-days before or 7 days after intra-venous injection of MDA-MB-231-IV cells and culled 28 days after tumour cell injection. Protocol outline shown in **a. b.** Shows the number +/− SEM and photomicrographs of DiD labelled, non-proliferating, tumour cells in the trabecular area of the tibia. **c.** Shows mean +/− SEM from two separate experiments (n=8 mice/experiment) percentage of mice with tumours in their hind limbs on day 28 and *ex vivo* photographs of GFP expressing tumours in the left tibiae of mice from experiment 2. Mean tumour area +/− SEM and time until detection of tumour growth in bone are shown in panels **d** and **e.** * = p < 0.01 compared with placebo, ^ = P < 0.01 compared with placebo or treatment protocol.

To investigate the role of IL-1 on breast cancer growth in bone we used two protocols outlined in Figure 1A: 1) A preventative protocol, in which 1mg/kg anakinra was injected every 24h for 31 days starting 3-days before injection of tumour cells to determine whether blocking IL-1R modified tumour cell homing and colonisation of bone; 2) a treatment protocol, in which 1mg/kg anakinra was injected every 24h for 21 days, starting 7 days after injection of tumour cells, to assess the effects on progression of established tumours. Pre-treatment with anakinra for 3-days had no effect on the numbers of disseminated tumour cells in bone (Figure 1b). Two-photon analysis showed similar numbers of DiD-labelled tumour cells present in the proximal tibiae of mice administered placebo or anakinra (treatment protocol or preventative protocol) 28 days following injection of tumour cells.

Daily injection of anakinra resulted in significantly fewer mice having detectable tumours in the long bones 28 days following injection of MDA-MB-231-IV cells (Figure 1c): Tumours were detectable in long bones of 80% of placebo treated mice, 38% of mice in the treatment group and 18% of mice in the preventative group (P = 0.04 and P = 0.01 compared with placebo for treatment and preventative, respectively) (Figure 1c). Administration of anakinra not only reduced numbers of mice with detectable tumours but also decreased tumour load. Tumour area was reduced by 62% in mice receiving anakinra using the treatment protocol and 91% in mice receiving the preventative protocol group (P > 0.001 and P > 0.0001 compared with placebo for treatment and preventative, respectively, n = 16 mice/group) (Figure 1d). Interestingly, administration of anakinra did not affect the time to detection of tumour in bones (Figure 1e); the first tumours were detected on day 19 in placebo treated mice, day 16 in mice administered anakinra under the treatment protocol and day 22 under the preventative protocol. These results suggest that blocking IL-1R signalling did not delay development of metastases from disseminated tumour cells, but decreased their capability to progress to overt metastatic tumours and/or the rate at which these tumours grew.

**Figure 2:**
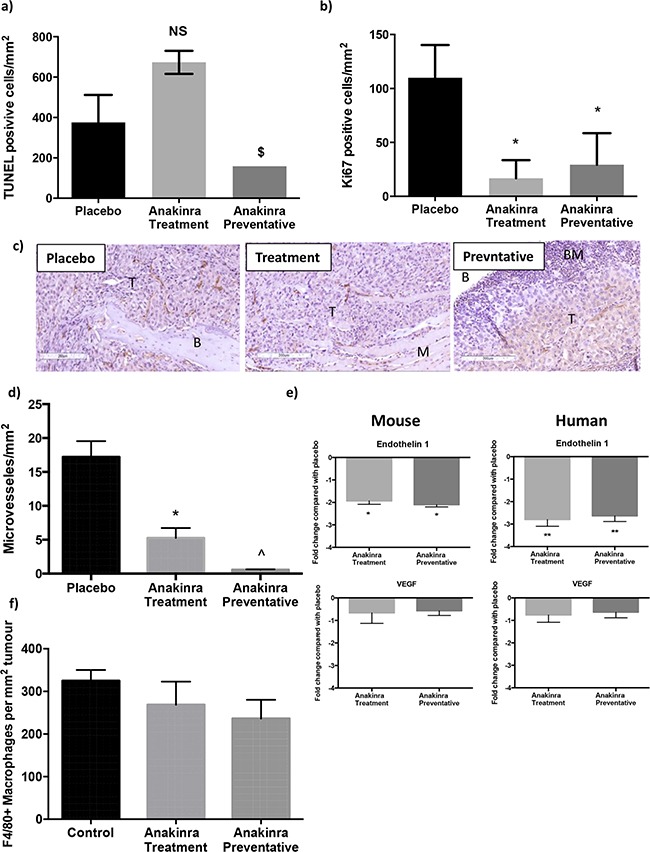
Effects of anakinra on viability of MDA-MB-231-IV tumours in bone Numbers of tumour cells undergoing apoptosis per mm^2^ tumour (+/− SEM) were analysed following in situ hybridisation using a TUNEL kit **a.** and numbers of actively proliferating cells per mm^2^ (+/− SEM) were assessed following Ki67 staining **b.** Photomicrographs show CD34 staining of endothelial cells in MDA-MB-231-IV tumours (T) in bone (B) 25 days after tumour cell injection **c.** numbers of CD34 positive blood vessels per mm^2^ of tumour are shown in (c) and **d.** shows Endothelin 1 and VEGF gene expression in mouse bone and human MDA-MB-231-IV tumours growing in mouse bone following daily injection with anakinra compared with placebo. Panel **f.** shows numbers of F4/80+ve macrophages per mm^2^ of tumour. * = p < 0.01 compared with placebo, ^ = P < 0.01 compared with placebo or treatment protocol.

Histological analysis of tumours in bone showed no statistical differences in numbers of TUNEL positive cells per mm^2^ of tumour between mice administered placebo those that received anakinra. However, tumours in bones from mice treated with the preventative anakinra protocol had significantly lower numbers of TUNEL positive cells (189 +/− 8.1 cells per mm^2^ of viable tumour) compared with tumours from mice receiving the treatment protocol (673 +/− 52 cells per mm^2^ of viable tumour) (P = 0.02) (Figure 2a). These data suggest that reduced tumour growth in the anakinra treated mice was not caused by increased levels of apoptotic tumour cell death. Administration of anakinra did have significant effects on tumour cell proliferation (Figure 2b) and angiogenesis (Figure 2c). Numbers of Ki67 positive tumour cells reduced by 85% in mice receiving anakinra via the treatment protocol and 73% in mice administered the preventative protocol, compared with placebo (P > 0.01 for treatment and preventative compared with placebo). Anakinra also significantly reduced the number of micro vessels in MDA-MB-231-IV bone tumours by 62% (treatment protocol) and 87% (preventative protocol), (P < 0.001 compared with placebo; Figure 2d). The anti-vascular effects of anakinra observed on histological sections were confirmed by gene expression analysis of Endothelin 1 and VEGF in tumours isolated from mouse tibiae. Both mouse and human Endothelin 1 mRNA levels was decreased 2 fold and 2.8 fold respectively following administration of anakinra via the treatment or preventative protocols compared with placebo (P < 0.01; Figure 2e). Repeated administration of a higher dose of anakinra, 10mg/kg, have previously been shown to increase macrophage infiltration into tumours [24]. In our current study 1mg/kg/day anakinra did not affect macrophage infiltration into tumours within bone (Figure 2f). Numbers of F480+ve cells reduced from 325.2 ± 25.17 per mm^2^ of tumour in the placebo group to 268.6 ± 54.39 per mm^2^ in the treatment group and 235.9 ± 44.00 per mm in the preventative group, no significant differences were recorded. Taken together, these data indicate that blocking IL-1R signalling does not kill tumour cells via apoptosis, but reduces the growth of tumours in bone via decreasing tumour cell proliferation and neo-vascularisation.

### Effects of anakinra on the bone microenvironment

**Figure 3:**
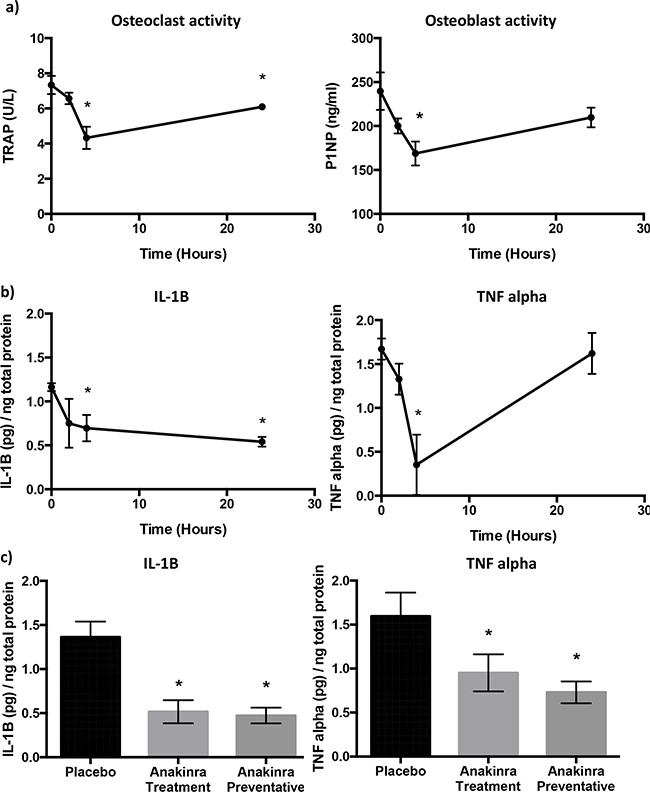
Effects of inhibition of IL-1R signalling on bone cell activity and cytokines ELISA analysis of protein lysates of whole mouse bone. Bone turnover was measured by TRAP (osteoclast activity) and P1NP (osteoblast activity) 0, 2, 4 and 24h after administration of a single dose of 1mg/kg anakinra **a.** Concentrations of pro-inflammatory cytokines IL-1B, and TNF alpha 0, 2, 4 and 24h after administration of a single dose of 1mg/kg anakinra are shown in panel **b.** Concentrations of IL-1B and TNF alpha in placebo and mice treated with 1mg/kg/day anakinra for 24 or 31 days are shown in panel **c.** Data shown are mean +/− SEM from 5 mice per group. * = p < 0.05.

We first investigated the effects of blocking IL-1R activity with a single 1mg/kg dose of anakinra on bone turnover and pro-inflammatory cytokines in bone marrow of BALB/c mice over a 24h time period (Figure 3). ELISA analysis shows a 41% drop in osteoclast (TRAP) activity over the first 4 hours (P = 0.02), osteoclast activity partially recovered over the next 20 hours to 6.10 +/− 0.17 U/L but remained 17% lower than the baseline recording of 7.33 +/− 0.52 U/L. Decreased osteoclast activity was mirrored by a decrease in osteoblast activity. P1NP concentrations decreased from 239.67 +/− 21.40 at baseline to 168.67 at 4 hours (P = 0.04). Similarly to osteoclasts, osteoblast activity partially recovered by 24h to 209.66 +/− 11.26 and although this remained 13% below the baseline levels there was no significant difference between P1NP concentrations at baseline and 24h following treatment (Figure 3a).

We next investigated whether blocking activity of IL-1R affected the secretion of pro-inflammatory cytokines into the bone marrow (Figure 3b). A single injection of anakinra resulted in decreased concentrations of IL-1B by 50%, from 1.162 +/− 0.04 pg/ng protein at baseline to 0.69 +/− 0.15 pg/ng protein 4h post injection and 0.54 +/− 0.05 pg/ng protein 24h after injection (P < 0.01 and P < 0.001 compared with 0h, respectively). Concentrations of TNF alpha also decreased by 80% from 1.67 +/− 0.12 pg/ng protein at baseline to 0.35 +/− 0.34 pg/ng protein 4h after injection, however, TNF alpha levels returned to baseline, 24h post injection with anakinra. Concentrations of IL-6 in the bone marrow of 6-week old BALB/c mice were low and did not change following administration of anakinra (data not shown).

To assess the effects of continuous treatment with 1mg/kg/day anakinra on pro-inflammatory cytokines in immunocompromised mice used in the tumour studies, bone marrow was taken from non-tumour bearing forelimbs and analysed 24h after administration of the last treatment (Figure 3c). Administration of anakinra for 24 days (treatment) or 31 days (preventative) reduced IL-1B from 1.36 ± 0.17 pg/ng of total protein (placebo) to 0.52 ± 0.13 pg/ng (P<0.01) and 0.47 ± 0.09 pg/ng of protein respectively (P <0.01). TNFα was also reduced from 1.60 ± 0.26 pg/ng of total protein in bone marrow from mice administered placebo to 0.95 ± 0.21 pg/ng protein in mice administered anakinra for 24 days (P < 0.01) and to 0.73 ± 0.13 pg/ng protein in mice administered anakinra for 31 days (P < 0.001). These data indicate that daily administration of anakinra leads to a sustained reduction in IL-1B and TNFα concentrations and is therefore an appropriate treatment strategy for investigating the effects if IL-1 on tumour growth in bone.

**Figure 4:**
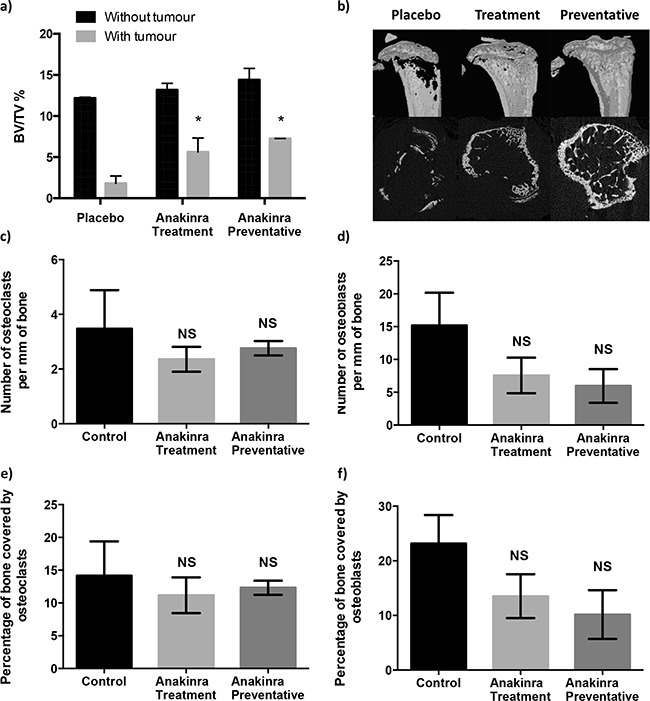
Effects of continuous IL-1R inhibition with anakinra on bone Histogram showing mean +/− SEM bone volume/tissue volume (BV/TV)% for trabecular bone in tibiae with and without tumours **a.** and uCT images of tumour bearing tibiae from mice treated with placebo or 1mg/kg anakinra/day using the treatment or preventative protocol **b.** Numbers of osteoclasts **c.** and osteoblasts **d.** lining trabecular bones and percentage of bone covered in osteoclasts **e.** and osteoblasts **f.** are from tumour bearing tibiae of mice treated with placebo or anakinra using the treatment or preventative protocols. Data shown are (mean +/− SEM), * = P < 0.01 compared with placebo.

Continued administration of anakinra for 21 days (in the treatment protocol) or for 31 days (in the preventative protocol) had no significant effect on trabecular bone volume in tumour-free tibiae (Figure 4a and 4b). In tumour bearing tibiae, administration of anakinra resulted in significantly increased bone volume: tissue volume (BV/TV) % compared with bones from mice administered placebo (P < 0.01 for the treatment and P < 0.001 for the preventative protocol). Injection of anakinra using either protocol showed a trend towards deceased numbers of osteoclast/osteoblasts per mm of trabecular bone and decreased percentage of trabecular bone in the tibiae covered by these cells compared with placebo, however, these differences were not statistically significant (Figure 4c-4f).

### Effects of anakinra on subcutaneous MDA-MB-231-IV and MCF7 tumours

**Figure 5:**
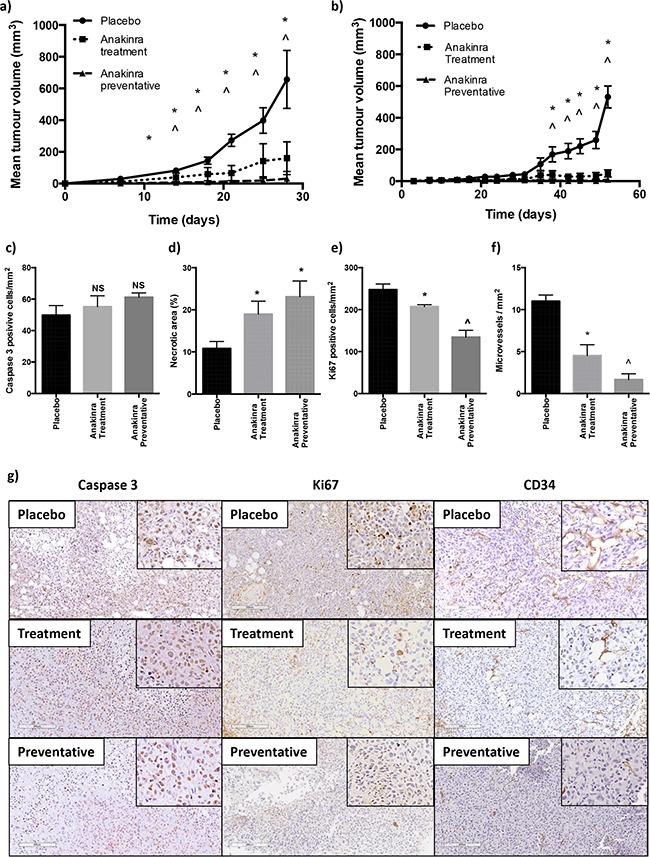
Effects of anakinra on subcutaneous MDA-MB-231-IV and MCF7 tumours MDA-MB-231-IV or MCF7 cells were grown subcutaneously and mice treated with placebo or 1mg/kg/day anakinra using the treatment or preventative protocol. Graphs show tumour volume for MDA-MB-231-IV cells **a.** and MCF7 cells **b.** Scoring data is for MDA-MB-231 tumours, number of active caspase 3 positive cells per mm^2^ of tumour **c.** % of tumour that is necrotic **d.** number of Ki67 positive cells per mm^2^ tumour **e.** and number of CD34 positive vessels per mm^2^ tumour **f.** mean +/− SEM. Representative X20 photomicrographs of subcutaneous tumours following immunohistochemical staining for active caspase 3, Ki67 and CD34 are shown in panel **g.** * = p < 0.01 compared with placebo, ^ = P < 0.01 compared with placebo or treatment protocol.

We next determined whether the anti-tumour effects of anakinra were restricted to ER-ve breast tumours growing in bone, or whether peripheral tumour growth could also be inhibited. For these experiments, ER-ve MDA-MB-231-IV or ER+ve MCF7 cells were injected subcutaneously and mice treated with anakinra according to the protocols outlined in Figure 1a. Administration of anakinra using the treatment protocol significantly decreased growth of subcutaneous MDA-MB-231-IV tumours from day 14 (P<0.001) and of MCF7 tumours from day 38 compared with placebo (P < 0.0001), which was further reduced using the preventative protocol (P > 0.0001) (Figure 5a and 5b). MCF7 tumours isolated from the preventative group were too small to allow *ex-vivo* analysis, therefore the anti-tumour effects of anakinra were assessed in isolated MDA-MB-231-IV tumours: Tumour cell apoptosis, assessed following immunohistochemistry for active Caspase 3, was not altered following daily injection with anakinra (Figure 5c and 5g), however necrotic cell death was significantly increased (P < 0.01 for treatment and P<0.001 for preventative compared with placebo) (Figure 5d). Proliferation was significantly reduced with tumours from placebo treated mice containing 247.8 +/− 29.25 Ki67 positive cells per mm^2^, this was reduced in tumours from mice receiving anakinra via the treatment protocol to 207.21 +/− 10.38 cells per mm^2^ (P < 0.01) and to 134.65 +/− 36.08 in tumours from mice administered anakinra using the preventative protocol (P < 0.001) (Figure 5e and 5g). In accordance with the decreased angiogenesis induced by anakinra in MDA-MB-231-IV tumours growing in bone, histological analysis of tumour sections stained for the endothelial cell marker CD34 showed reduced angiogenesis in subcutaneous MDA-MB-231-IV tumours from mice that had received daily anakinra compared with placebo. Compared with placebo, the number of CD34 positive microvessels in subcutaneous tumours decreased by 81% following administration of anakinra via the treatment protocol (P < 0.01) and by 94% following the preventative protocol (P < 0.001) (Figure 5f-5g).

These data show that inhibition of IL-1R signalling with anakinra significantly reduces subcutaneous growth of both ER+ve and ER-ve tumours and analysis of MDA-MB-231-IV derived tumours support that this is mediated by altering the tumour microenvironment, increasing tumour cell necrosis and reducing proliferation and angiogenesis.

## DISCUSSION

The primary aim of this study was to investigate the effects of blocking IL-1B activity through inhibition of the IL-1R, on breast cancer bone metastasis. Our data show that daily administration of the IL-1R antagonist anakinra significantly reduces development and progression of breast cancer bone metastases. This did not appear to be a result of reduced ability of MDA-MB-231-IV cells to locate to bone as originally hypothesised [1]. It is well established that following dissemination of MDA-MB-231 cells in bone only a small number (>0.1%) proliferate into overt metastasis whilst the rest remain dormant [25]. Labelling MDA-MB-231-IV cells with DiD before injection enabled quantification of tumour cells that had disseminated in the bone environment and remained dormant throughout the experimental period [23, 25–26]. Pre-treatment with anakinra 3-days before inoculation of tumour cells had no effect on the number of non-proliferative cells detected in bone, therefore, it is likely that administration of anakinra did not affect tumour cell dissemination in bone. Instead, our data indicate that inhibition of IL-1R with anakinra inhibits proliferation of tumour cells within bone, holding these in a dormant state and subsequently preventing progression to metastases. The increased anti-tumour effects observed with the preventative protocol compared with the treatment protocol may be due in part to the extended time period in which anakinra can exert an effects (31 vs. 21 days) in addition to micrometastases already being present in mice from the treatment protocol before the administration of anakinra. Anakinra reduced the numbers of mice in which tumours developed into overt metastases, not by directly killing tumour cells through induction of apoptosis, but by reducing tumour cell proliferation and angiogenesis. Our data suggest that these anti-tumour effects are a result of changes in the tumour microenvironment, as direct incubation of MDA-MB-231-IV cells with anakinra did not exert anti-tumour affects *in vitro* (Supplementary Figure S1). The bone is rich in remodelling cytokines, including TNF alpha, IL-1B and IL-6, and previous studies have reported that incubation of MDA-MB-231 cells with a combination of these cytokines in a 3D model system stimulates their proliferation [24]. In our study, administration of anakinra significantly reduced expression of TNF alpha and IL-1B in bone (Figure 3) and reduced levels of these molecules is likely to have contributed to the anti-tumour effects observed.

Bone metastases are traditionally associated with increased bone turnover. It is generally accepted that breast cancer cells growing in bone release growth factors that stimulate osteoclastic bone resorption and the release of growth factors from bone that, in turn, stimulate growth of the tumour; a process is known as the vicious cycle [28]. Based on this theory and evidence from mouse model systems, we hypothesise that increasing bone turnover would result in increased metastases from tumour cells disseminated in this site and current clinical practise as well as our previous work has focussed on inhibiting bone resorption to treat bone metastases [18, 22–23, 29]. Removal of IL-1B or IL-1R1 in mouse knockout models has profound effects on the bone microenvironment, resulting in reduced bone resorption, increased trabecular thickness and skeletal growth, suggesting that silencing of central IL-1R signalling leads to progressive accrual of bone mass [30–31]. In the current study, anakinra significantly reduced osteoclastic bone resorption and osteoblastic bone formation within 4h of administration, these returned to near normal levels by 24h when the next dose was administered. Overall, this dosing regime led to a small reduction in the number of osteoclasts or osteoblasts on the bone surface and although there was a trend towards increased bone volume this did not reach significance (Figures 3 and 4). It is, therefore, possible that anti-tumour effects of anakinra may partially be the result of reduced bone turnover, however, it is more likely that the substantially lower bone tumour burden observed in mice treated with this agent is the result of inhibition of tumour cell proliferation and angiogenesis.

Vascular endothelial cells secrete pro-inflammatory cytokines, including IL-1B and TNF alpha [32], with increased expression of the latter linked with increased expression of endothelins [33]. In accordance with previously published data showing reduced primary tumour growth and CD34 positive vasculature in 4T1 mammary tumours treated with anakinra [24], our study also showed that inhibition of MDA-MB-231-IV tumour growth in bone following administration of anakinra was associated with reduced levels of CD34 positive blood vessels. Further analysis revealed that daily administration of anakinra significantly reduced endothelin 1 gene expression in both mouse bone and human tumours. These data imply that there is a feedback between cytokine production and development of new endothelial cells, and that blocking IL-1B and TNF alpha production with anakinra reduces the formation of new blood vessels, thereby starving the tumour of nutrients and oxygen. In contrast to previous studies showing increased VEGF expression in endothelial cells treated with IL-1B [34] and reduced IL-1B and TNF alpha activity in bone marrow mononuclear cells stimulated with VEGF [35], we did not see a significant decrease in VEGF gene expression in either mouse bone or MDA-MB-231-IV tumours growing in bone following administration of anakinra, compared with placebo. We know that VEGF signalling is complex and tumour heterogeneity as well as redundancy in the VEGF signalling system result in resistance to anti-VEGF therapies in cancer patients including those with breast cancer [Reviewed in: 36–37]. The role that cytokines like IL-1B play in this redundancy remains to be established.

The anti-tumour effects of anakinra were not confined to tumours in bone. This may indicate that alterations to the bone microenvironment are not the sole reason for reduced tumour growth, however, we cannot discount the possibility that anakinra may affect vascular precursors within the bone marrow; further investigations are required to address this question. Daily treatment of mice with anakinra starting 7 days after subcutaneous injection of MDA-MB-231-IV or MCF7 cells significantly reduced tumour growth which was further reduced when mice were pre-treated with anakinra for 3-days before tumour cell implantation. As with MDA-MB-231-IV cells growing in bone, reduced tumourigenesis was not due to an increase in cell death by apoptosis but an increase in tumour cell necrosis, reduction in tumour cell proliferation and angiogenesis. These data suggest that anakinra causes changes in the subcutaneous tumour microenvironment, possibly via reduced IL-1B and TNF alpha signalling on endothelial cells preventing neo-angiogenesis and reducing nutrient/oxygen supply to the growing tumours. Our findings are in agreement with previously published studies showing that treatment of subcutaneous MCF7 tumours with a combination of tamoxifen, flaxseed and eterolactone resulted in production of a naturally occurring IL-1 receptor antagonist from the tumours and these tumours exhibited reduced growth and angiogenesis [38]. Similarly, studies in mouse models of Lewis Lung and 4T1 mammary cancer, showed decreased growth of primary tumours following administration of paclitaxel, anakinra or a combination of the two [24]. In this study, administration of paclitaxel increased secretion of IL-1B and subsequent development of lung metastases, leading the authors to hypothesise that increased IL-1B was driving metastasis. However, this study subsequently reported that administration of a high dose (10mg/kg) anakinra for 3 consecutive days starting 24h before paclitaxel (25 or 50mg/kg) did not inhibit metastasis. Using this high dose of anakinra appeared to increase lung metastasis, increase leakage from blood vessels and increase numbers of tumour-associated macrophages [24]. It is important that decreased bone metastasis seen following administration of anakinra is not replaced by increased lung metastasis. In our current study, we used bone seeking MDA-MB-231-IV cells, these cells rarely metastasise to lung (~2%) and daily treatment with 1mg/kg anakinra did not induce lung metastasis. Furthermore, using this lower dose of anakinra did not affect numbers of tumour-associated macrophages found in bone metastases. It is possible that the lack of T cells our mouse models is the reason that we did not see increased lung metastasis following anakinra and the role of the immune system warrants further investigation. It should also noted that anakinra has a half life of 4-6 h [39] and concentrations of IL-1B spike to above background levels 48h after withdrawal of high doses of this antagonist (3-10mg/kg) [40], therefore, increased IL-1 after anakinra withdrawal may promote lung metastasis in the Lewis lung and 4T1 models. Withdrawal of clinically relevant doses of anakinra (1mg/kg/day) used in the current study is not reported to induce a spike in circulating IL-1B, indicating that this dose may be more effective for treating breast cancer metastasis. Further investigations are needed to establish whether continuous treatment with anakinra is necessary for sustained inhibition of metastasis.

Anakinra inhibits the IL-1R-mediated signalling by both IL-1B and IL-1A. We cannot be sure to what extent the observed anti-tumour effects are due to reduced IL-1B signalling. It is possible that inhibition of IL-1A, as well as downstream effects on other cytokines including TNF alpha, contribute to the reduced of growth of both subcutaneous and bone tumours following administration of anakinra. There are a number of reports showing the pro-tumourigenic effects of TNF alpha in breast cancer [reviewed in 41]. However, independent of the mechanism by which anakinra reduces growth of MCF7 and MDA-MB-231-IV breast tumours, blocking IL-1R may be of therapeutic benefit to breast cancer patients. Anakinra (up to 100mg/day; equivalent to the 1 mg/day in mice used in this study) is licenced to treat patients with neonatal onset multi system inflammatory disease and rheumatoid arthritis [13–14]. Anakinra is generally well tolerated and severe side effects (neutropenia and hepatotoxicity) are rare, the main disadvantage being the requirement for daily injections. Clinical trials of anakinra in breast cancer to investigate the clinical significance of our findings could therefore be initiated relatively easily.

In conclusion, our data provide the first evidence that continuous inhibition of IL-1 activity inhibits breast tumour growth and progression to bone metastasis *in vivo.* Inhibition of IL-1R with a clinically relevant dose of anakinra significantly alters the tumour and bone microenvironments leading to reduced tumour cell proliferation and angiogenesis and increased tumour cell necrosis as well as reduced bone turnover. These findings indicate that IL-1 may provide a potential novel therapeutic target for breast cancer bone metastasis.

## MATERIALS AND METHODS

### Cell culture

MCF7 cells (European Collection of Authenticated Cell Cultures) and an eGFP expressing bone homing derivative of MDA-MB-231 produced in house, MDA-MB-231-IV [1] were maintained in RPMI-1640 supplemented with 10% FCS (Gibco Invitrogen, Paisley UK). Both cell lines were authenticated, in house, using short tandem repeat analysis of 10 loci. Prior to *in vivo* inoculation, eGFP expressing cells were incubated for 15 minutes with 25μM of 1,1′-Dioctadecyl-′, 3′-Tetramethylindodicarbocyanine, 4-Chlorobenzenesulfonate (DiD) (Life Technologies, Paisley, UK) to enable detection of disseminated tumour cells in bone by two-photon microscopy. Tumour growth was monitored *in vivo* using an Illumatool Lighting System (LightTools Research, Encintas, CA) (eGFP).

#### *In vitro* studies

Cell proliferation was monitored every 24h for 144 hours by manual cell counting using a 1/400^2^ haemocytometer (Hawksley, Lancing, UK). Tumour cell invasion was assessed using 6mm Transwell plates with an 8.0μM pore size (Costar; Corning Incorporated, NY, USA) coated with basement membrane matrix (20% Matrigel; Invitrogen, Paisley, UK). Cells were seeded into the inner chamber at a density of 2.5×10^5^ for MDA-MB-231-IV and 5×10^5^ for MCF7 in RPMI without FCS and 5×10^5^ HS5 bone marrow cells in RPMI supplemented with 10% FCS were added to the outer chamber. Cells were removed from the top surface of the membrane 24 and 48 hours after seeding, and cells that have invaded through the pores were stained with haematoxylin and eosin before being imaged on Leica DM7900 light microscope and manually counted.

Migration of cells was investigated by analysing wound closure: Cells were seeded onto 0.2% gelatine in 6-well tissue culture plates (Costar; corning incorporated); once confluent, 10ug/ml mitomycin C was added to inhibit cell proliferation and a 50uM scratch made across the monolayer. The percentage of wound closure was measured 24 and 48 hours using a CTR7000 inverted microscope and LAS-AF v2.1.1 software (Leica Applications Suite; Leica Microsystems, Wetzlar, Germany).

#### *In vivo* studies

Tumour growth studies used 6-week-old female BALB/c nude mice (13-16g) to enable modelling of bone metastasis. To investigate the effects of anakinra on circulating cytokines we used BALB/c wild-type and immunocompromised mice (Charles River, Kent, UK). Experiments were carried out in accordance with local guidelines and with Home Office approval under project licence 40/3462, University of Sheffield, UK.

To investigate the effects on serum levels of IL-1B, TNF alpha and IL6, 1mg/kg anakinra (r-metHu1L-ra, equivalent to 100mg currently licenced to treat rheumatoid arthritis) or placebo (control) (Amgen, Cambridge, UK) was injected subcutaneously (s.c) into 20 mice. Mice were culled 0, 2, 4 and 24h after injection. Bone marrow and serum were collected and stored at -80°C prior to analysis.

To assess the effects of on initiation of tumour development in soft tissue and in bone, mice were pre-treated with 1mg/kg/day anakinra or placebo starting 3-days before injection of 5×10^6^ MCF7 or 0.5×10^6^ MDA-MB-231-IV cells s.c. or intra-venously (i.v.) via the lateral tail vein (n=8/group/experiment). To investigate whether anakinra reduces growth of established tumours in soft tissue and bone, 0.5×10^5^ MDA-MB-231-IV cells were injected s.c. or i.v. (n=8/group/experiment) 7 days prior to commencement of anakinra treatment. Each experiment was repeated twice and mice injected with MDA-MB-231-IV cells were culled 35 days (experiment 1) or 28 days (experiment 2) after tumour cell injection. Mice injected with MCF7 cells were supplemented with 4mg/L B-estradiol (Sigma Aldrich, Poole, UK) via their drinking water and culled 56 days after tumour cell injection.

Serum was stored at -80°C for ELISA, tibiae and femurs were fixed in 4% PFA for microcomputed tomography (μCT) analysis before decalcification in 1%PFA/0.5% EDTA and processing for histology. Bones for two-photon analysis were stored in OCT at -80°C.

### Microcomputed tomography imaging

μCT analysis was carried out using a Skyscan 1172 x-ray-computed μCT scanner (Skyscan, Aartselar, Belgium) equipped with an x-ray tube (voltage, 49kV; current, 200uA) and a 0.5-mm aluminium filter. Pixel size was set to 5.86 mm and scanning initiated from the top of the proximal tibia as previously described [18].

### Bone histology and measurement of tumour volume

Subcutaneoustumour volume was measured twice per week using vernier callipers. Bone tumour areas were measured on3, non-serial, H&E stained,5mM histological sections of decalcified tibiae per mouse using Osteomeasure software (Osteometrics inc. Decauter, USA) and a computerised image analysis system.

Osteoclasts were detected by tartrate-resistant acid phosphatase (TRAP) staining as previously described [19]. Osteoblasts were identified as mononuclear, cuboidal cells residing in chains along the bone surface. The number of osteoclasts/osteoblasts per mm of cortical-endosteal bone surface and trabecular bone surfaces and the proportion of bone surface occupied by osteoclasts/osteoblasts was determined using a Leica RMRB upright microscope and OsteoMeasure software as previously described [20].

### Immunohistochemistry and in situ hybridization

Immunohistochemistry for caspase-3 was performed using a rabbit polyclonal antibody that specifically recognizes active mouse caspase-3 (AF835, 1:750 dilution; R&D Systems, Minneapolis, MN), followed by a biotin–conjugated anti-rabbit secondary antibody (1:200 dilution; Vector Laboratories, Peterborough, UK.) as described by Marshman *et al*. [21]. Immunohistochemistry for the cell proliferation antigen Ki-67 was carried out as previously described [22] using a mouse monoclonal antibody specific for human Ki-67 (MIB-1, 1:125 dilution; DakoCytomation, Cambridge, UK) followed by a biotin–conjugated anti-mouse secondary antibody (1:200;Vector Laboratories). The endothelial cell antigen CD34 and macrophage specific antigen F4/80 were detected as described for Ki-67 with the use of a rat monoclonal antibodys specific for mouse CD34 (MCA1825GA, 1:50 dilution; Serotec, Oxford, UK) or mouse F4/80 1:100 dilution; BioRad, Oxford, UK) followed by a biotin–conjugated anti-rat secondary antibody (E0467, 1:200 dilution; DakoCytomation). In situ hybridization to detect cells in the early stages of apoptosis was performed using a Millipore ApopTag® Peroxidase in situ Apoptosis detection kit (TUNEL) and manufacturers instructions (Merck Millipore, Durham, UK).

Necrosis, apoptosis and proliferation were scored on two stained histological sections per tumor sample. % Area of necrotic tumour was measured following H&E staining and numbers of active caspase-3–positive, TUNEL positive or Ki-67–positive cells innon-necrotic tumourwere counted with the use of an Leica BMRB upright microscope and OsteoMeasure software. Quantification of microvasculature was established by quantifying the mean number of CD34 positive stained blood vessels through a Chalkley grid count. Briefly, a 25-point grid was placed over a 20X magnification lens of a Leica RNRB upright microscope; all vellels touching the grid were counted. 5 randomly selected fields of non-necrotic tumour were scored on 2 non-serial sections per tumour.

### Two-photon microscopy

Tibiae were imaged using a multiphoton confocal microscope (LSM510 NLO upright; Zeiss, Cambridge, UK). DiD labelled cells were visualised using a 900nm Chameleon laser, bone was detected using the 633nm multiphoton laser (Coherent, Santa Clara, CA.) and images were reconstructed in LSM software version 4.2 (Zeiss) as previously described [23].

### Biochemical analysis

Serum concentrations of TRACP-5b and P1NP were measured using commercially available ELISA kits: MouseTRAP™ Assay (Immunodiagnostic Systems) and Rat/Mouse P1NP competitive immunoassay kit (Immunodiagnostic Systems), respectively. Concentrations of IL-1B, TNF alpha and IL-6 were analysed in mouse bone marrow and serum using anti-mouse Quantikine ELISA kits (R&D systems, Abingdon, UK) and manufacturers protocols.

### Real-time PCR

Mouse and human VEGF and endothelin 1 gene expression was analysed on cDNA isolated from tumour-bearing mouse bones using Taqman assays (VEGF: Hs00900055_m1 (human) Mm01281449 (mouse); endothelin 1: Hs00174961 (human) Mm00438656 (mouse)) (all reagents from Applied Biosystems, Warrington UK): Relative gene expression compared with the housekeeping gene glyceraldehyde-3-phosphate dehydrogenase (GAPDH; Hs99999905_m1) was assessed using an ABI 7900 PCR System (Perkin Elmer, Foster City, CA) and Taqman universal master mix). Fold change in gene expression between treatment groups was assessed by directly inserting CT values into Data Assist V3.01 software (Applied Biosystems) and changes in gene expression were only analysed for genes with a CT value of ≤25.

### Statistical analysis

Statistical analysis was by unpaired T-test except for Kaplan-Meier survival charts for which one tailed Mantel-Hansel and log-rank tests for trend were used. All analysis were carried out using GraphPad PRISM® software version 6.0. Statistical significance was defined as P less than or equal to 0.05.

## SUPPLEMENTARY FIGURE


