# Supplementary material for: IL-1 drives breast cancer growth and bone metastasis in vivo
Source: Oncotarget. 2016 Sep 27;7(46):75571–84. doi: 10.18632/oncotarget.12289 (PMC5342762; doi:10.18632/oncotarget.12289)
Supplement: Supplementary file 1 [file oncotarget-07-75571-s001.pdf]

IL-1 drives breast cancer growth and bone metastasis *in vivo*

SUPPLEMENTARY FIGURE

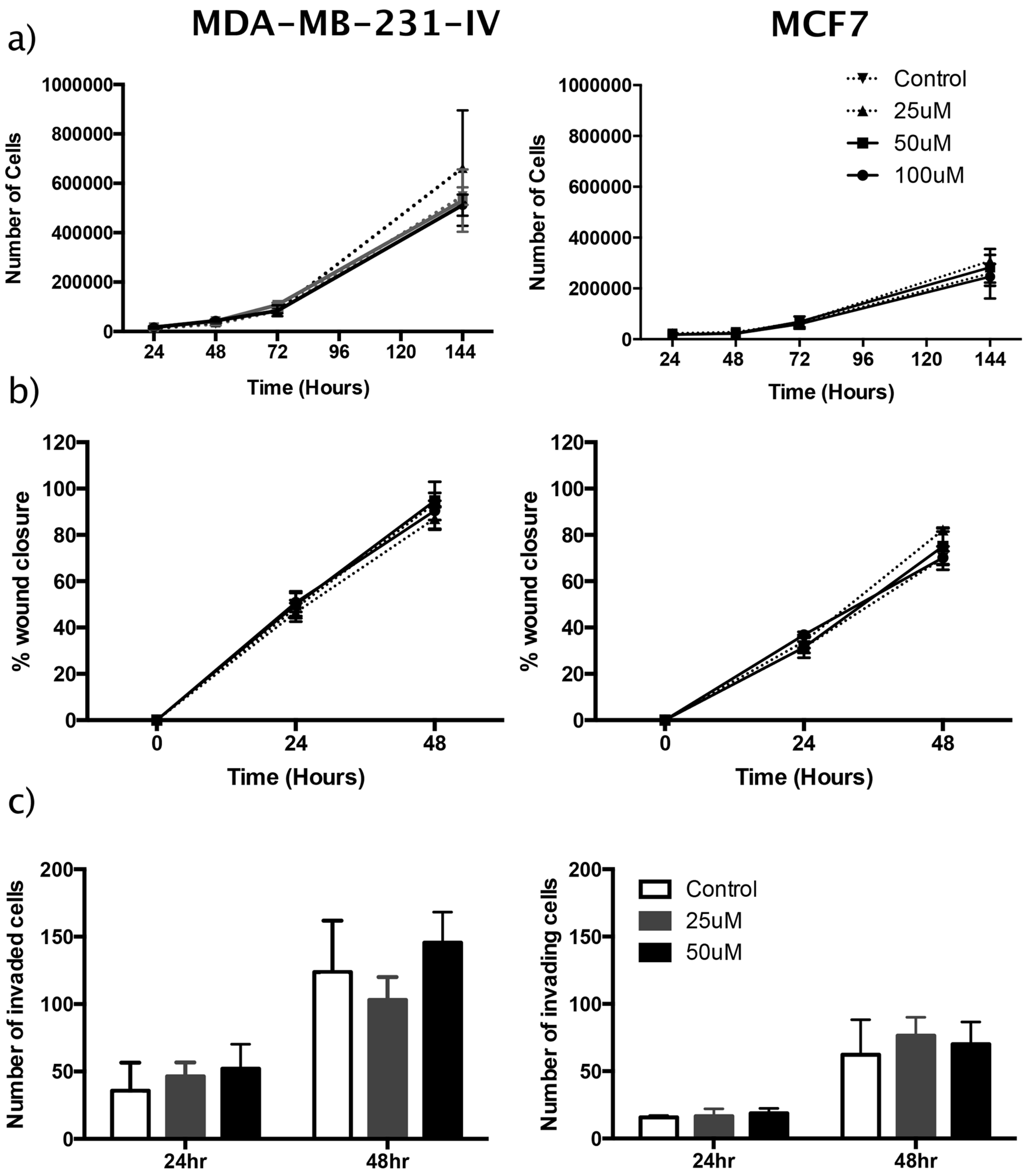

Supplementary Figure S1: Anti-tumour effects of anakinra on MDA-MB-231-IV and MCF7 cells *in vitro*. Effects of placebo, 25, 50 and 100uM anakinra on proliferation of MDA-MB-231-IV and MCF7 Cells were assessed 1-144h after cell seeding **a.** Effects on migration were assessed using a wound closure assay 24 and 48 hours after administration of placebo, 25, 50 and 100uM anakinra **b.** and numbers of cells that invaded through a matrigel coated Transwell plates were measured 24 and 48h after administration of placebo, 25 or 50uM anakinra. All graph show mean +/- SD of the data.
